# Do predictors of abstinence change in the medium- and long-term follow-up of smokers who have quit smoking? A prospective cohort study

**DOI:** 10.18332/tpc/208884

**Published:** 2025-11-03

**Authors:** José I. de Granda-Orive, Carlos A. Jiménez-Ruiz, Maria Isabel Cristóbal-Fernández, Carlos Rábade-Castedo, Paz Vaquero-Lozano, Elia Pérez-Fernández, María Inmaculada Gorordo-Unzueta, Lourdes Lázaro-Asegurado, Eva de Higes-Martínez, Juan Antonio Riesco-Miranda, Rosa Mirambeaux-Villalona, Gloria Francisco-Corral, Alejandro Frino-García, Jaime Signes-Costa Miñana, Cristina Villar-Laguna, Ana María Cicero-Guerrero, Julio Cesar Vargas-Espinal, Teresa Peña-Miguel, Jacobo Sellares, Ángela Ramos-Pinedo

**Affiliations:** 1 Medicine Department, 12th October University Hospital, Complutense University of Madrid, Madrid, Spain; 2 San Carlos Clinical Hospital, Madrid, Spain; 3 University Clinical Hospital of Santiago de Compostela, La Coruña, Spain; 4 Hermanos Sangro Community Health Center, Gregorio Marañón General University Hospital, Madrid, Spain; 5 Alcorcón Foundation University Hospital, Madrid, Spain; 6 Respiratory Department, Galdakao-Usansolo University Hospital, Galdakao, Spain; 7 Respiratory Department, Burgos University Hospital, Burgos, Spain; 8 Respiratory Department, University Hospital of Cáceres, Cáceres, Spain; 9 Respiratory Department, Ramón y Cajal University Hospital, Madrid, Spain; 10 Respiratory Department, Mancha Centro University General Hospital, Alcázar de San Juan, Spain; 11 Pulmonology Department, Hospital Clínic de Barcelona, Barcelona, Spain; 12 Respiratory Department, University Clinical Hospital of Valencia, Valencia, Spain

**Keywords:** smoking cessation, predictors, tobacco dependence, smoking, quitting predictor, multivariate regression

## Abstract

**INTRODUCTION:**

We hypothesize that the predictors of smoking cessation in the medium-term are not the same as in the long-term of follow-up. The aim of this study was to identify predictors for smoking cessation (continuous abstinence) and determine if these are maintained over time.

**METHODS:**

This is an observational longitudinal (prospective cohort) multicenter study conducted in daily clinical practice in Spain. Patients were consecutively enrolled as they attended consultations, and all patients followed for 12 months. To identify predictors of smoking cessation (at 24 and 52 weeks post-cessation) we have collected sociodemographic and clinical data, smoking consumption characteristics, and psychological and physical dependence variables. Multivariate logistic regression models were fitted. The analysis was by intention to treat.

**RESULTS:**

A total of 337 participants were considered for the study. Predictors of smoking cessation at 24 weeks were baseline weight (AOR=1.02; 95% CI: 1–1.03), not having made a previous quit attempt (AOR=2.72; 95% CI: 1.44–5.15), lower sedation levels on the psychological dependence test (AOR=1.78; 95% CI: 1.06–2.97), and adherence to treatment (AOR=8.03; 95% CI: 3.85–16.73). At 52 weeks, predictors of smoking cessation were being male (AOR=2.38; 95% CI: 1.35–4.18), low self-efficacy (AOR=2.60; 95% CI: 1.36–5.00), not having made a previous quit attempt (AOR=5.06; 95% CI: 2.20–11.66), lower sedation levels on the psychological dependence test (AOR=1.96; 95% CI: 1.13–3.40), and adherence to treatment (AOR=12.03; 95% CI: 4.14–34.94). These last three predictors were those that were maintained between 24 and 52 weeks of follow-up.

**CONCLUSIONS:**

Not having previous attempts to quit smoking, lower sedation levels in the psychological dependence test, and having greater adherence to treatment have been maintained as predictors of quitting over time.

## INTRODUCTION

Smoking is a leading cause of preventable death^1^. Promoting reduced tobacco use is a simple and cost-effective measure to reduce premature death and disability^2,3^. One of the key measures to reduce the demand for tobacco use is that tobacco users need help to quit^4^. So, it is important to establish effective measures to promote cessation of tobacco use and adequate treatment for tobacco dependence^5,6^. The problem is that tobacco dependence is not easy to quit. The efficacy of various methods of smoking cessation becomes a vital area of research. Various strategies, including behavioral and pharmacological, as well as a combination of both, have shown different levels of efficacy in smoking cessation, but it is known that a combination of them emerged as the most successful approach to quitting smoking^7,8^. The effectiveness of these interventions is influenced by a multitude of factors, including genetic predisposition, individual characteristics, aspects directly related to tobacco consumption, and socioeconomic status^4^. Acknowledging these factors is important when tailoring interventions to diverse demographic groups^7^.

Therefore, one of the most interesting and studied aspects of achieving better results in quitting smoking is knowing the predictors of quitting. For many years, several previous studies have investigated predictors of smoking cessation attempts and continued abstinence. Numerous predictors of smoking cessation have been included previously and at different times after quitting^9-12^. The motivation to quit smoking as a predictor of attempts and abstinence has studies for and against^13^. It has been reported that a low level of dependence and a high level of self-efficacy have been shown to be predictors of abstinence after the attempt to quit^13,14^.

Some research^15^ has found that if the participants can maintain abstinence for a period of 24 weeks, they are very likely to maintain long-term abstinence for a period of 52 weeks. But previous studies have also suggested that the factors that help a smoker quit initially may not be the same as those that help him/her remain abstinent in the long-term^15,16^.

We hypothesize that the predictors of smoking cessation in the medium-term are not the same as in the long-term of follow-up. The aim of this study was to identify predictors for smoking cessation (continuous abstinence) at 24 and 52 weeks post-cessation and determine if these are maintained over time among smokers treated in a multicenter study conducted in daily clinical practice in Spain.

## METHODS

### Design

The observational longitudinal (prospective cohort) multicenter study was conducted in smoking clinics in daily clinical practice in Spain in seven tertiary hospitals, two secondary hospitals, and a community-specialized smoking unit. Patients were consecutively enrolled as they attended consultations from 1 December 2023 to 31 December 2024, and all patients followed for 12 months. An estimate of the number of patients to be treated was made, identifying 325 patients for an estimated annual abstinence rate with pharmacological treatment of 30% with a confidence level of 95% (assuming a level of error of 5% with respect to the abstinence rate estimate).

This study adhered to the Strengthening the Reporting of Observational Studies in Epidemiology (STROBE) checklist for observational research (Supplementary file SF1).

### Inclusion and exclusion criteria

The inclusion criteria were: smoking patients motivated to quit smoking who agree to participate in the study and sign informed consent. The exclusion criteria were: 1) aged <18 years and >65 years; 2) contraindication for the use of cytisine (hypersensitivity to the active ingredient or to any of the excipients including mannitol, microcrystalline cellulose, magnesium stearate, glycerol, cybehenate, hypromellose), unstable angina, recent history of myocardial infarction (previous 4 weeks), clinically relevant arrhythmias (previous 4 weeks), recent history of cerebrovascular accident (previous 4 weeks), and pregnancy and breastfeeding; 3) patients with renal insufficiency; 4) patients with hepatic insufficiency; and 5) patients with psychiatric illness.

### Ethical approval and informed consent

The study was conducted in accordance with the principles of the Declaration of Helsinki and in accordance with Good Clinical Practice guidelines. The information was processed in compliance with Regulation (EU) 2016/679 of the European Parliament and of the Council of 27 April 2016, on the protection of natural persons with regard to the processing of personal data and on the free movement of such data, following the recommendations and instructions issued by the Spanish Data Protection Agency. Smoking patients assessed during smoking consultations were informed about the nature of the study and were asked to participate and sign informed consent. This work was presented and approved by each one of the ethics committees of the participating centers (number of our centre: N^o^ CEIm: 22/300).

### Collected variables

*Main outcome*

The main outcome was continuous abstinence at 24 and 52 weeks of follow-up, validated by cooximetry (exhaled air CO levels <10 ppm were required to consider a subject a non-smoker)^17^.

*Predictor factors*

Variables considered to assess their impact on continuous abstinence at 24 and 52 weeks were collected by *ad hoc* questionnaire and validated measure questionaries:

- Sociodemographic and clinical data (all qualitative variables except for weight): age, sex, race, education level (no formal education or secondary education, higher education), employment status (unemployed, pre-retired, or retired; actively employed; student; or homemaker), residence (rural, urban), baseline weight, comorbidities such as presence of cardiovascular diseases (hypertension), pulmonary diseases (chronic obstructive pulmonary disease and asthma), neoplasia, and drugs.
- Smoking consumption characteristics: years of smoking, age at smoking initiation, current number of cigarettes per day (categorized as ≥20 cigarettes/day), pack-years (categorized as ≥15 pack-years), previous quit attempts ( ‘previous attempts’, ‘previous attempts in the last year’, ‘previous attempts with drug treatment’ and ‘previous attempts with varenicline’ and graded as mild, moderate, severe or very severe) and smoking cessation treatments, degree of smoking (mild, moderate, severe, or very severe), and baseline exhaled CO levels (ppm).
- Psychological dependence variables: motivation to quit smoking and perceived self-efficacy [assessed using Visual Analog Scales (0–10), and categorized as ≥8], type of reinforcement according to the Fagerström Reinforcement Questionnaire (negative, smoking to relieve withdrawal symptoms; positive, smoking for pleasure; or both factors)^18^.
- Physical dependence: according to the Fagerström test for nicotine dependence (FTND) with a cut-off of ≤6 points and time to first cigarette after waking (<30 minutes or <5 minutes)^19^.
- Psychological dependence: according to the test from the Specialized Smoking Unit of the Madrid Health Department, UIPSM)^18^. This questionnaire measures 7 domains and each domain analyzed using the cutoff points described in the literature: stimulation, ≥11; automatism, ≥11; sedation, ≥6; social dependence, ≥14; psychological dependence, ≥7; and gestural dependence, ≥14. The different subscales are defined as: 1) stimulation (stimulate oneself) to make someone want to do something or do it more (Items 1 to 4); 2) sedation (sedate oneself) by smoking to pacify, soothe, or calm down. It is a negative reinforcement that is a learning process where a behavior is strengthened by the removal of an unpleasant or aversive stimulus (Items 5 and 6); 3) automatism is an involuntary behavior or actions performed without conscious awareness or control behind an addictive drug (Items 7 to 10); 4) social dependence is an association that a smoker establishes between tobacco consumption and specific social situations, routines or environments (Items 11 to 15); 5) psychological dependence is an association of smoking with specific emotional states, situations, or activities, creating a conditioning that leads the smoker to seek out a cigarette in certain circumstances (smoker’s preoccupation with tobacco) (Items 16 to 18); 6) gestural dependence is the ritual of lighting a cigarette, taking a puff then putting it out, which becomes a habit that is hard to break (everyday situations associated with smoking) (Items 19 and 23); and 7) treatment adherence at 4 weeks, defined as the degree to which the behavior of a patient, in relation to taking medication (with checking medication withdrawals at the community pharmacy), corresponds to the recommendations agreed with the healthcare professional (<50%, 50–80%, >80%).

In Supplementary file SF2 we have captured the different scales used (in Spanish).

### Smoking cessation interventions: procedures

We defined abstinence as ‘continuous abstinence’^20^. We consider continuous abstinence when the subject refrains from smoking, from the moment they stop smoking until the end of the follow-up, as validated by co-oximetry. The treatment and follow-up intervention to stop smoking followed the regulations in force with a known protocol^21^. The clinical-psychological care protocol for the treatment of smoking with cytisine included a total of eight visits, of which one was the baseline consultation and the remaining seven were follow-up consultations. Of these seven follow-up consultations, three were carried out over the 25 days in which the subject was using the medication. The patient attended the baseline visit consultation in person. The first check-up is carried out 6 to 8 days after starting the medication, in person and individually. The second check-up is carried out between 16 and 18 days, and the third between 24 and 26 days after starting to take the medication, both in person. The remaining check-ups are carried out at 8 and 12 weeks and at 6 and 12 months, all of them in person. The duration of the first visit was 40 minutes, and in the follow-up visits 15 minutes. Smokers were treated in each clinic by its staff (doctor, nurse, and psychologist were added in some clinics).

### Statistical analysis

Data were analyzed using STATA 17 (Stata Corp. 2017. Stata Statistical Software: Release 15. College Station, TX: Stata Corp LLC). Categorical variables are presented as frequencies and percentages, while quantitative variables are summarized as mean and standard deviation (SD) or median and interquartile range (IQR), depending on data distribution. We conducted a univariate analysis to explore differences in characteristics based on successful smoking cessation at 12 months. The chi-squared test was used for categorical variables, while the t-test or Mann-Whitney U test was applied for two independent samples of quantitative variables.

To identify predictors of smoking cessation, univariate and multivariate logistic regression models were fitted. In multivariate models, variable selection was performed using the adaptive Lasso (Least Absolute Shrinkage and Selection Operator)^22^ method, a penalized regression approach that assigns variable-specific weights, allowing for more efficient selection of relevant predictors. This approach enhances model interpretability, mitigates multicollinearity, and reduces overfitting by shrinking less important coefficients toward zero while retaining significant variables. The shrinkage parameter (*λ*) was determined via 10-fold cross-validation (CV) using the minimum criteria, which selects the *λ* value that minimizes the cross-validation error. To visualize this process, we plotted the CV error function against different values of *λ*, identifying the optimal point where prediction error is minimized. Additionally, a coefficient path plot was generated to examine how regression coefficients evolved as *λ* increased. This graphical representation illustrates the progressive shrinkage of coefficients, with less relevant predictors approaching zero first, while stronger predictors remain in the model. This helps assess the stability of selected variables and ensures that only meaningful predictors contribute to the final model. Lost to follow-up patients at the 4th week were considered non-adherent and patients who did not attend follow-up visits despite attempts at new appointments have been considered as no response.

Model coefficients are reported as adjusted odds ratios (AORs) with corresponding 95% confidence intervals (CIs), and the area under the receiver operating characteristic curve (ROC-AUC) was used to evaluate the model’s discriminatory ability in distinguishing between successful and unsuccessful smoking cessation cases. Statistical significance was set at p≤0.05.

## RESULTS

### Characteristics of participants

**Figure 1:**
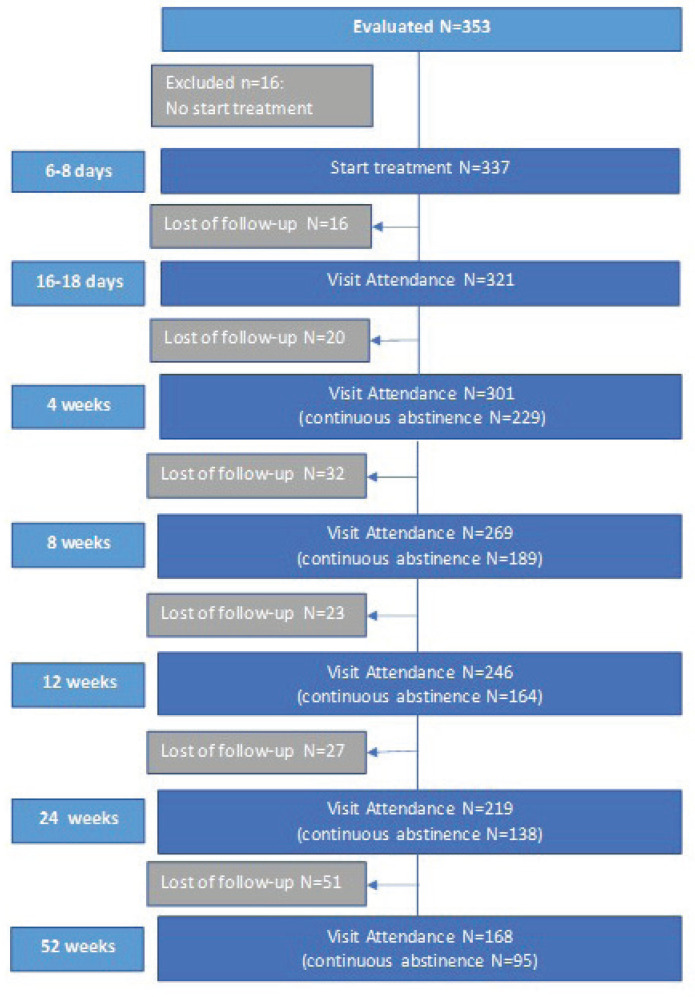
Participant flowchart, observational longitudinal (prospective cohort) multicenter study, Spain (N=337)

A total of 353 participants were enrolled in the smoking cessation program, but 16 subjects did not start treatment. Finally, 337 participants were considered for the study, of which 118 (35.01%) were lost at 24 weeks of follow-up, while 219 (64.98%) were maintained in the study (the analysis was by intention to treat, so the 337 total participants were included in the analysis). Between 24 weeks and one year of follow-up, another 51 patients were lost, so 168 (49.85%) patients finally completed treatment and follow-up (95 patients were in continuous abstinence) (Figure 1). The participants had an average age of 54.2 (SD=9.1) years, 51% were women, and 60% of the patients had some comorbidity. Regarding smoking characteristics, 70% had severe smoking, 54% had a high degree of nicotine dependence (FTND ≥7), 84% had high motivation (VAS ≥8) to make a quit attempt, but only 31% reported high self-efficacy (VAS ≥8), and 83% of patients had made previous attempts (Table 1).

**Table 1:** Characteristics of the participants, observational longitudinal (prospective cohort) multicenter study, Spain (N= 337)

*Characteristics*	*Categories*	*Total*		*Abstinence at 24 weeks*					*Abstinence at 52 weeks*				
				*No*		*Yes*		*p*	*No*		*Yes*		*p*
		*N=337*		*N=199*		*N=138*			*N=242*		*N=95*		
		*n*	*%*	*n*	*%*	*n*	*%*	*n*	*%*	*n*	*%*		
**Age** (years), mean (SD)		54.2	9.1	53.0	9.6	55.8	8.2	**0.006**	53.6	9.4	55.5	8.4	0.095
**Sex**	Male	164	49.0	88	44.4	76	55.5	**0.047**	109	45.2	55	58.5	**0.029**
**Race**	Black	1	0.3	1	0.5			1.000	1	0.4			1.000
	White	328	98.5	193	98.5	135	98.5		235	98.3	93	98.9	
	Latino	4	1.2	2	1.0	2	1.5		3	1.3	1	1.1	
**Education level**	Higher	155	46.1	89	44.9	66	47.8		116	4.81	39	41.1	
**Employment status**	Unemployed/pre-retired/retired	86	25.6	44	22.2	42	30.4	0.090	59	24.5	27	28.4	0.456
**Residence**	Urban	292	86.6	167	83.9	125	90.6	0.077	209	86.4	83	87.4	0.807
**Baseline weight** (kg), mean (SD)		75.6	0.2	73.6	0.2	78.5	0.2	**0.009**	53.6	9.4	55.5	8.4	0.166
**Comorbidities**		203	60.2	119	59.8	84	60.9	0.843	149	61.6	54	56.8	0.425
	Cardiovascular diseases	103	30.6	56	28.1	47	34.1	0.246	75	31.0	28	29.5	0.785
	Hypertension	61	18.1	31	15.6	30	21.7	0.149	44	18.2	17	17.9	0.951
	Pulmonary diseases	117	34.7	75	37.7	42	30.4	0.169	90	37.2	27	28.4	0.128
	COPD	53	15.7	32	16.1	21	15.2	0.831	41	16.9	12	12.6	0.328
	Asthma	32	9.5	24	12.1	8	5.8	0.054	27	11.2	5	5.3	0.097
	Neoplasia	24	7.1	12	6.0	12	8.7	0.349	14	5.8	10	10.5	0.128
	Drugs	34	0.1	22	11.1	12	8.7	0.479	25	10.3	9	9.5	0.814
**Years smoking**, median (IQR)		36	28–42	33	25–40.5	39	30–44	**0.002**	35	27–41	39	30–44	**0.027**
**Age of smoking initiation**, median (IQR)		16	15–18	16	15–18	17	15–18	0.738	16	15–18	17	15–18	0.887
**Cigarettes/day**	≥20	267	79.5	158	79.4	109	79.6	0.971	196	81.0	71	75.5	0.266
**Pack-years**	≥15	318	94.9	186	93.5	132	97.1	0.141	225	93.8	93	97.9	0.168
**Previous quit attempt**		275	83.3	167	86.1	108	79.4	0.109	202	85.6	73	77.7	0.081
	Last year previous attempt (N=275)	73	26.5	55	32.9	18	16.7	**0.003**	66	32.7	7	9.6	**<0.001**
	Pharmacological treatment (N=275)	153	55.6	95	56.9	58	53.7	0.604	118	58.4	35	47.9	0.123
	Varenicline (N=275)	89	32.4	55	32.9	34	31.5	0.801	69	34.2	20	27.4	0.290
**Degree of smoking**	Severe/very severe	237	70.3	139	69.8	98	71.0		168	69.4	69	72.6	
**Motivation**	≥8	282	84.4	161	82.1	121	87.7	0.169	199	83.3	83	87.4	0.351
**Self-efficacy**	≥8	102	30.8	68	35.2	34	24.6	**0.040**	85	36.0	17	17.9	**0.001**
**Reinforcement** (FRQ)	Negative (relieve withdrawal symptoms)	161	48.2	93	47.4	68	49.3	0.576	118	49.4	43	45.3	0.064
	Positive (pleasure)	92	27.5	58	29.6	34	24.6		71	29.7	21	22.1	
	Both	81	24.3	45	23.0	36	26.1		50	20.9	31	32.6	
**Baseline CO levels** (ppm), median (IQR)		18	13–25	18	13–25	17	12–24	0.242	18	13–25	16	12–23	0.080
**Physical dependence**	FTND ≥7	181	53.9	107	54.0	74	53.6	0.940	135	56.0	46	48.4	0.208
	TFG ≥30	294	87.5	176	88.9	118	85.5	0.356	212	88.0	82	86.3	0.680
	TFG ≥5	125	37.2	75	37.9	50	36.2	0.759	91	37.8	34	35.8	0.737
**Psychological dependence** (UIPSM)	Stimulation ≥11	23	6.9	12	6.1	11	8.0	0.500	17	7.1	6	6.4	0.813
	Automatism ≥11	19	5.7	11	5.6	8	5.8	0.930	14	5.9	5	5.3	0.849
	Sedation ≥ 6	203	61.0	135	68.9	68	49.6	**<0.001**	160	66.9	43	45.7	**<0.001**
	Social dependence ≥14	50	15.0	30	15.3	20	14.6	0.859	39	16.3	11	11.7	0.289
	Psychological dependence ≥7	189	56.8	122	62.2	67	48.9	**0.016**	148	61.9	41	43.6	**0.002**
	Gestural dependence ≥14	9	2.7	7	3.6	2	1.5	0.317	8	3.3	1	1.1	0.454
**Treatment adherence at 4 weeks**	>80%	249	73.9	120	60.3	129	93.5	**<0.001**	158	65.3	91	95.8	**<0.001**

IQR: interquartile range. COPD: chronic obstructive pulmonary disease. FRQ: Fagerström reinforcement questionnaire. FTND: Fagerström test for nicotine dependence. TFG: time to the first cigarette. UIPSM: Specialized Smoking Unit of the Madrid Health Department Questionnaire.

When we compared patient characteristics by response group at 24 weeks of follow-up, we found that abstinent participants were older (p=0.006) and more frequently men (p=0.047). Abstinent participants also had a higher mean weight (p=0.009), for every kg, the probability of abstinence increases by 2%. Regarding smoking-related variables, abstinent participants had been smoking for longer (p=0.002), had made fewer quit attempts in the past year (p=0.003), and exhibited lower self-efficacy (p=0.040). In terms of psychological dependence, abstinent individuals had significantly lower sedation scores than those who continued smoking (p<0.001). Furthermore, a higher percentage of abstinent participants had an adherence rate above 80% at the end of pharmacological treatment (4 weeks) compared to non-abstinent participants (93.5% vs 60.3%, p<0.001) (Table 1).

At 52 weeks of follow-up, no statistically significant differences in age or baseline weight were found; however, the difference about sex remained statistically significant (p=0.029). Similar to the 24-week findings, abstinent participants had been smoking for longer (p=0.027), had made fewer quit attempts in the past year (p<0.001), and reported lower self-efficacy (p=0.001). Lower sedation levels among abstinent individuals persisted as a significant factor (p<0.001). Moreover, adherence rates >80% at the end of the pharmacological treatment were again associated with higher abstinence rates (95.8% vs 65.3%, p<0.001) (Table 1).

### Successful smoking cessation predictors

**Figure 2:**
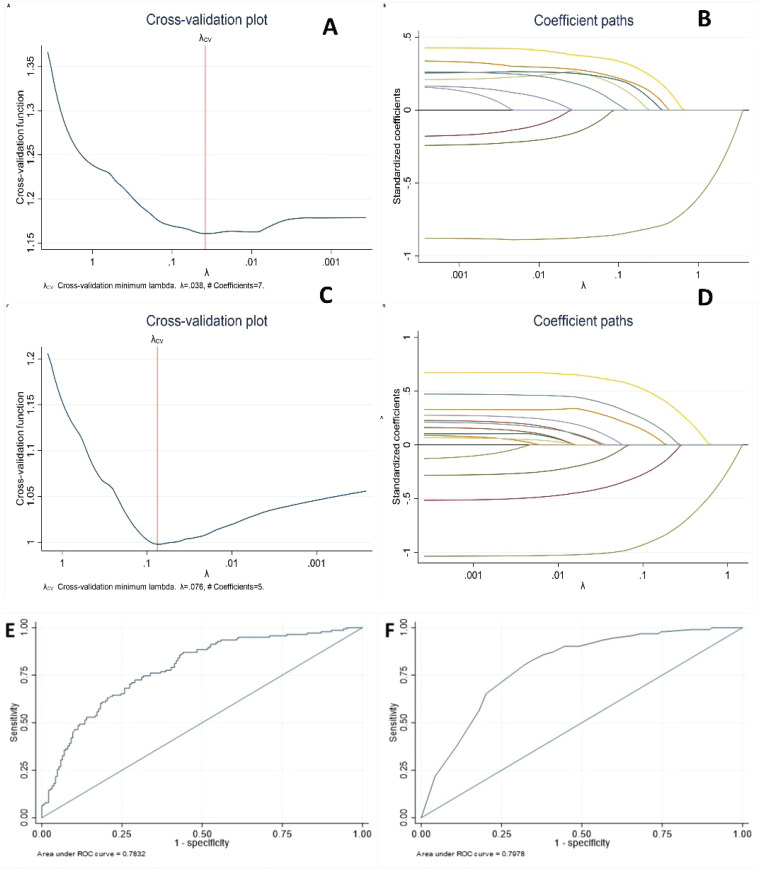
Cross-validation (CV) and coefficient paths plots of predictor selection LASSO procedure at 24 weeks (A, B) and 52 weeks (C, D). Cross-validation presents CV error function against different values of lambda, identifying the optimal point where prediction error is minimized. Coefficient path plot represents the progressive shrinkage of coefficients as lambda increased, with less relevant predictors approaching zero first, while stronger predictors remain in the model. Final multivariate model’s performance in distinguishing between participants who achieved abstinence and those who did not at 24 weeks (E) and 52 weeks (F). Observational longitudinal (prospective cohort) multicenter study, Spain (N=337)

LASSO regression was conducted to identify potential predictors among 23 candidate variables. For the 24-week follow-up, seven predictors were selected with a *λ*=0.038: weight, smoking years, previous attempt, motivation, self-efficacy, sedation (item in the psychological dependence test), and adherence (Figures 2A and 2B). Multivariate regression analysis at 24 weeks, incorporating these predictors, revealed statistically significant effects for weight (AOR=1.02; 95% CI: 1–1.03), not having made a previous quit attempt in the last year (AOR=2.72; 95% CI: 1.44–5.15), lower sedation levels on the psychological dependence test (AOR=1.78; 95% CI: 1.06–2.97), and adherence to treatment (AOR=8.03; 95% CI: 3.85–16.73). Smoking years, age and self-efficacy did not reach statistical significance, but they were retained as contributing predictors according to the LASSO criterion. The final model had an acceptable to good performance with ROC-AUC=0.783. (Table 2 for 24 weeks of follow-up, and Figure 3E).

**Table 2:** Impact of continuous abstinence at 24 and 52 weeks of predictor factor studied, observational, longitudinal (prospective cohort), multicenter study, Spain (N=337)

*Predictor*	*Category*	*Univariate logistic regression*				*Multivariate logistic regression*			
		*OR*	*p*	*95% CI*		*AOR*	*p*	*95% CI*	
				*Upper*	*Lower*			*Upper*	*Lower*
**At 24 weeks of follow-up**									
**Age** (years)		1.03	**0.008**	1.01	1.06				
**Sex**	Male	1.37	0.153	0.89	2.12				
**Education level**	Higher	1.05	0.832	0.68	1.62				
**Employment status**	Unemployed/pre-retired/retired	1.46	0.135	0.89	2.38				
**Baseline weight** (kg)		1.02	**0.012**	1.00	1.03	1.02	**0.017**	1.00	1.03
**Comorbidities**	Yes	1.11	0.641	0.71	1.73				
**Smoking years**		1.04	**0.001**	1.01	1.06	1.03	0.064	1.00	1.05
**Pack-years**	≥15	2.40	0.134	0.76	7.52				
**Previous year quit attempt**	No	2.53	**0.002**	1.42	4.49	2.72	**0.002**	1.44	5.15
**Degree of smoking**	Severe/very severe	1.11	0.657	0.69	1.79				
**Motivation**	≥8	1.46	0.229	0.79	2.71	1.89	0.077	0.93	3.82
**Self-efficacy**	0–7	1.75	**0.024**	1.08	2.85	1.68	0.079	0.94	2.98
**Reinforcement**	FRQ negative	1.35	0.231	0.83	2.21				
**Physical dependence**	FTND ≤6	1.05	0.832	0.68	1.62				
	TFG ≥30	1.30	0.428	0.68	2.49				
	TFG ≥ 5	1.08	0.739	0.69	1.69				
**Psychological dependence** (UIPSM)	Stimulation ≥11	1.55	0.311	0.66	3.62				
	Automatism ≥11	1.26	0.629	0.50	3.18				
	Sedation ≤5	2.22	**0.001**	1.41	3.48	1.78	**0.029**	1.06	2.97
	Social dependence ≤13	1.22	0.530	0.66	2.26				
	Psychological dependence ≤6	1.69	**0.019**	1.09	2.63				
	Gestural dependence ≤13	2.60	0.239	0.53	12.69				
**Treatment adherence at 4 weeks**	>80%	8.66	**0.000**	4.28	17.50	8.03	**0.000**	3.85	16.73
**At 52 weeks of follow-up**									
**Age** (years)		1.02	0.096	1.00	1.05				
**Sex**	Male	1.71	**0.030**	1.05	2.77	2.38	**0.003**	1.35	4.18
**Education level**	Higher	0.75	0.242	0.46	1.21				
**Employment status**	Unemployed/pre-retired/retired	1.22	0.457	0.72	2.09				
**Baseline weight** (kg)		1.01	0.167	1.00	1.02				
**Comorbidities**	Yes	0.82	0.425	0.51	1.33				
**Smoking years**		1.03	**0.022**	1.00	1.06				
**Pack-years**	≥ 15	3.10	0.138	0.70	13.82				
**Previous year quit attempt**	No	4.14	**0.000**	1.90	9.01	5.06	**0.000**	2.20	11.66
**Degree of smoking**	Severe/very severe	1.17	0.562	0.69	1.98				
**Motivation**	≥8	1.39	0.352	0.69	2.78				
**Self-efficacy**	0–7	2.58	**0.002**	1.43	4.65	2.60	**0.004**	1.36	5.00
**Reinforcement**	FRQ negative	1.49	0.162	0.85	2.60				
**Physical dependence**	FTND 0–6	1.36	0.209	0.84	2.18				
	TFG ≥30	1.16	0.680	0.57	2.34				
	TFG ≥5	1.09	0.737	0.66	1.78				
**Psychological dependence** (UIPSM)	Stimulation ≥	0.89	0.813	0.34	2.33				
	Automatism ≥	0.90	0.849	0.32	2.58				
	Sedation ≤5	2.40	**<0.001**	1.48	3.91	1.96	**0.016**	1.13	3.40
	Social dependence ≤13	1.47	0.291	0.72	3.01				
	Psychological dependence ≤6	2.10	**0.003**	1.30	3.41				
	Gestural dependence ≤13	3.22	0.273	0.40	26.11				
**Treatment adherence at 4 weeks**	>80%	12.09	**<0.001**	4.29	34.07	12.03	**0.000**	4.14	34.94

FRQ: Fagerström Reinforcement Questionnaire. FTND: Fagerström test for nicotine dependence. TFG: time to the first cigarette. UIPSM: Specialised Smoking Unit of the Madrid Health Department Questionnaire. AOR: adjusted odds ratio.

For the 52-week follow-up, five predictors were selected with a λ=0.076: sex, previous quit attempt, self-efficacy, sedation, and adherence (Figures 2C and 2D). Multivariate regression analysis at 52 weeks showed statistically significant effects for being male (AOR=2.38; 95% CI: 1.35–4.18), not having made a previous quit attempt in the last year (AOR=5.06; 95% CI: 2.20–11.66, p<0.001), low self-efficacy (AOR=2.60; 95% CI: 1.36–5), lower sedation levels on the psychological dependence test (AOR=1.96; 95% CI: 1.13–3.40), and adherence to treatment (AOR=12.03; 95% CI: 4.14–34.94). The final model had an acceptable to good performance with ROC-AUC=0.798 (Table 2 for 52 weeks of follow-up, and Figure 2F).

## DISCUSSION

Numerous studies have sought predictors of long-term success in quitting smoking. In general, the following predictors of smoking cessation are repeated in all studies: being male, being older, living with a partner, being pregnant, having a high income, low alcohol consumption, lower daily cigarette consumption and previous smoking time, lower rate of exhaled carbon monoxide at the beginning, low nicotine dependence, later time to smoke the first cigarette in the morning after getting out of bed, no smoking cigarettes after the first few days of quitting, starting smoking after the age of 20 years, having several previous quit attempts, not having other smokers around at home or at work, having higher self-efficacy at the beginning, better management of skills, strong decision to quit, use of pharmacological treatment in the attempt, not being exposed to secondhand smoke, having higher motivation, availability of cigarettes, high adherence to the cessation treatment program, if the price of tobacco increases, family and friends support, restrictions on smoking at home, fear of the effects of tobacco, doing sports daily and those who use technologies such as mobile phone applications^9-16^.

We found statistically significant predictors of quitting smoking at 24 weeks to be higher mean weight initially, not having previously attempted to quit smoking, having a lower score in those who smoke to sedate themselves, and having greater adherence to treatment. At 52 weeks, we found that the significant predictors of quitting were being male, having lower self-efficacy at baseline, again having no prior quit attempts, lower sedation levels on the psychological dependence test, and having higher adherence to treatment. So, we found that some predictors of quitting smoking were maintained in the follow-up.

In our study, some of the predictors of success at 24 weeks are the same as those found at 52 weeks. Liao et al.^15^ also found some predictors of success that persisted between weeks 24 and 52, and if the participants can maintain abstinence for a period of 24 weeks, they are very likely to maintain long-term abstinence for a period of 52 weeks. But this does not seem to be the norm in monitoring abstinence over time after quitting smoking. There is some evidence that factors measured during treatment, as opposed to baseline, are more predictive of long-term abstinence^16^. Bailey et al.^16^ conclude that predictors of initial induction of change (24-hour withdrawal) were not predictors of abstinence at one year, suggesting that different factors mediate the different subprocesses of behavior change. The majority of studies have focused on factors that affect maintenance over the medium- to long-term, paying much less attention to factors that might control the induction of the initial treatment. Maintenance of change is the ultimate test of treatment effectiveness, but for those who are unable to sustain even short periods of abstinence, maintenance is a non-issue. Findings of these two studies^15,16^ suggest that the factors that help a smoker quit initially may not be the same as those that help him/her remain abstinent long-term. We must understand the factors that influence the initial phase of quitting precisely because so many smokers have great difficulty achieving a successful quit attempt^23^. On the other hand, according to the study by Jackson et al.^24^, which analyzed continuous abstinence rates over time, it is suggested that the factors affecting early relapse of smoking may differ from those driving later relapse. This could imply that the predictors of abstinence may be different at different stages after quitting smoking. Thus, Jackson et al.^24^ does not specifically identify different predictors of abstinence between weeks 24 and 52, the idea that relapse factors may vary over time suggests the possibility that abstinence predictors may also differ at different points in the smoking cessation process.

The strongest predictor of abstinence in our work, both at 24 and 52 weeks, was adherence to treatment. In our study, treatment adherence referred to practically all cytisine intake. Al-Dahshan et al.^25^ identified adherence to drug treatment as one of the predictors of smoking cessation success at 6 months. They found that smokers who used varenicline, nicotine replacement therapy, or a combination of both for smoking cessation were more than twice as likely to quit smoking compared to those who did not use these medications^25^. Joo et al.^26^ found that the use of smoking cessation medications (varenicline or bupropion) was not significantly associated with long-term quitting. Conversely, the regular attendance at the smoking cessation program was significantly associated with successful quitting in both the short-term and long-term among our participants^26^. In the same sense, Hays et al.^27^ found that adherence to pharmacotherapy for smoking cessation is highly correlated with improved tobacco abstinence. There was a positive correlation between adherence to treatment and tobacco abstinence^27^.

Another powerful predictor of quitting in our work, both at 24 and 52 weeks, has been having no prior quit attempts in the last year. This is striking, since one of the predictors that has been repeated in the different studies has been having several previous attempts to quit smoking^9,28-30^. Recently, a study whose objective was to estimate the rate and predictors of smoking cessation at 6 months follow-up in smokers attending smoking cessation clinics in primary care settings^25^ found that smoking cessation was less likely among those who had a higher number of previous quit attempts. Other authors, previously, have also found having fewer previous quit attempts to be a predictor of abstinence^31,32^. It was predicted that people who had unsuccessful attempts would tend to lose confidence in themselves and see themselves as failures, which would stop them from quitting in the future. A failed quit attempt to stop smoking was found to be an indicator of subsequent relapse when compared to those with no recent attempt^33^. So, motivation and intent to quit might be indicators of quit attempts but not necessarily successful ones^13^. The role of habitual quitters who continuously try and fail was also highlighted in a prospective study^34^.

In our clinics, we use a psychological dependence test (test from the Specialized Smoking Unit of the Madrid Health Department, UIPSM) that, among other items, measures whether the smoker uses tobacco to sedate (calm down) themselves. We found that smokers who used tobacco less to calm down were more likely to remain abstinent at both 24 and 52 weeks. Many smokers believe that smoking reduces anxiety and stress, which in turn discourages some from quitting. However, smoking is actually known to increase anxiety and tension^4^. The feeling of stress reduction or relaxation is temporary and is soon replaced by withdrawal symptoms and cravings. It is known that smoking cessation was associated with an improvement in mental health symptoms compared with continuing to smoke (anxiety and depression symptoms, symptoms of stress, positive affect)^4^. Hence, our finding is consistent with this fact, as those who use tobacco less to sedate themselves will have a better chance of staying smoke-free.

Another predictor of abstinence added at 52 weeks of follow-up was lower self-efficacy before quitting smoking. In a previous study by our group, we found no association between self-efficacy measured at baseline and the probability of success at the end of follow-up, but in that study, we did not measure self-efficacy throughout the entire quit attempt. It is therefore striking that low self-efficacy was associated with successful abstinence at 52 weeks in this study, as previously published data indicate that it is a poor predictor of abstinence when measured before an attempt, but this association is somewhat more robust when measured after the day of quitting. Self-efficacy correlates positively with success in maintaining abstinence, but negatively with quit attempts^14^. Likewise, as in other studies^9,10^, being male was positioned as a predictor of abstinence at 52 weeks but not at 24 weeks.

### Limitations

The present work has several potential limitations. First, there were missing data when the patient did not attend any of the scheduled visits or there is a loss of follow-up. We have performed the analysis under a ‘worst-case scenario’ approach, imputing missing data in response as no abstinence. Second, the findings were obtained using smokers who voluntarily attended smoking cessation clinics, and the surveys were performed in different scenarios and geographical locations, which might not reflect the general population. Third, the use of questionnaires in patients is not always accurate. Fourth, the sampling strategy and the dimensions of the sample may not have sufficient statistical strength to identify differences. Fifth, numerous previous studies have found other variables associated with successful cessation. We did not analyze all of the previously analyzed variables, which undermines the precision of our study. Sixth, some confounding factors may not have been included in the analysis (residual confounding: the error or bias that remains in a study’s results even after attempts have been made to control for confounding variables) and there is the possibility of a ‘diagnostic bias’ on the part of the researchers who performed the smoking cessation measurements.

## CONCLUSIONS

We have found that some of the predictors of abstinence at 6 months are maintained at 52 weeks of follow-up; this would indicate a robust strength of these predictors of abstinence, as they are maintained in the medium- and long-term during the process of quitting smoking. We found as predictors of abstinence at 24 weeks of follow-up a higher mean weight initially, not having previously attempted to quit smoking, having a lower score in those who smoke to sedate themselves, and having greater adherence to treatment. At 52 weeks, we found that the significant predictors of quitting were being male, having lower self-efficacy at baseline, having no prior quit attempts, lower sedation levels on the psychological dependence test, and having higher adherence to treatment. These last three predictors were those that were maintained between 24 and 52 weeks of follow-up after quitting smoking. These findings give a framework for tailoring effective anti-smoking measures addressing adults.

## Supplementary Material



## Data Availability

The data supporting this research are available from the authors on reasonable request.
