# Erratum: Do predictors of abstinence change in the medium- and long-term follow-up of smokers who have quit smoking? A prospective cohort study

**DOI:** 10.18332/tpc/215501

**Published:** 2025-12-12

**Authors:** European Publishing Production Team

**Affiliations:** 1 European Publishing Production Team, Heraklion, Greece

**Keywords:** smoking cessation, predictors, tobacco dependence, smoking, quitting predictor, multivariate regression

**Erratum on:**

**Do predictors of abstinence change in the medium- and long-term follow-up of smokers who have quit smoking? A prospective cohort study**

**By José I. de Granda-Orive, Carlos A. Jiménez-Ruiz, Maria Isabel Cristóbal-Fernández, Carlos Rábade-Castedo, Paz Vaquero-Lozano, Elia Pérez-Fernández, María Inmaculada Gorordo-Unzueta, Lourdes Lázaro-Asegurado, Eva de Higes-Martínez, Juan Antonio Riesco-Miranda, Rosa Mirambeaux-Villalona, Gloria Francisco-Corral, Alejandro Frino-García, Jaime Signes-Costa Miñana, Cristina Villar-Laguna, Ana María Cicero-Guerrero, Julio Cesar Vargas-Espinal, Teresa Peña-Miguel, Jacobo Sellares, Ángela Ramos-Pinedo**

**Tobacco Prevention & Cessation, Volume 11, Issue November, Pages 1–16**

**Publish date: 3 November 2025**

DOI: https://doi.org/10.18332/tpc/208884

In the original article, in the sixteenth page, the **Disclaimer** section was missing.

It has been now corrected as follows:

## DISCLAIMER

C. A. Jiménez-Ruiz, Editorial Board member of the journal, had no involvement in the peer-review or acceptance of this article and had no access to information regarding its peer-review. Full responsibility for the editorial process for this article was delegated to a handling editor of the journal.

The mentioned changes are corrected also online.

