# Supplementary material for: Do predictors of abstinence change in the medium- and long-term follow-up of smokers who have quit smoking? A prospective cohort study
Source: Tob Prev Cessat. 2025 Nov 3;11:10.18332/tpc/208884. doi: 10.18332/tpc/208884 (PMC12581196; doi:10.18332/tpc/208884)
Supplement: Supplementary file 1 [file TPC-11-51-s1.pdf]

Supplementary File SF2. The different scales used in the study.

Motivation scale (Visual Analogue Scale)

MOTIVACIÓN PARA DEJAR DE FUMAR

Señale en esta escala cuál es su grado de motivación para dejar de fumar, suponiendo que 0 se corresponde con la ausencia total de motivación y 10 con sentirse plenamente motivado.

0 1 2 3 4 5 6 7 8 9 10

Perceived self-efficacy (Visual Analogical Scale)

EVALUACIÓN DE LA AUTO-EFICACIA

Señale en esta escala su capacidad para dejar de fumar, siendo 0 la ausencia total de capacidad y 10 la máxima.

0 1 2 3 4 5 6 7 8 9 10

Fagerström Reinforcement Questionnaire

TEST DE RECOMPENSA

Cuando usted ha intentado dejar de fumar, ¿cuál de estas dos circunstancias ha sido su mayor dificultad?

A) No poder consumir un cigarrillo en aquellas situaciones en las que realmente me apetecía y me hacía sentirme bien (Recompensa positiva).

B) Padecer de forma continua síntomas como: ansiedad, irritabilidad y nerviosismo. (Recompensa negativa).

Test de Fagerström

TEST DE FAGERSTRÖM

- ¿Cuánto tiempo pasa entre que se levanta y se fuma su primer cigarrillo?

– Hasta 5 minutos	3
– De 6 a 30 minutos	2
– De 31 a 60 minutos	1
– Más de 60 minutos	0

- ¿Encuentra difícil no fumar en lugares donde está prohibido (hospital, cine, biblioteca)?

– Sí	1
– No	0

- ¿Qué cigarrillo necesita más?

– El primero de la mañana	1
– Cualquier otro	0

- ¿Cuántos cigarrillos fuma al día?

– Menos de 10 cigarrillos/día	0
– Entre 11 y 20 cigarrillos/día	1
– Entre 21 y 30 cigarrillos/día	2
– 31 ó más cigarrillos	3

- ¿Fuma con más frecuencia durante las primeras horas después de levantarse que durante el resto del día?

– Sí	1
– No	0

- ¿Fuma aunque esté tan enfermo que tenga que guardar cama la mayor parte del día?

– Sí	1
– No	0

Puntuación total .... [ ]

Test from the Specialised Smoking Unit of the Madrid Health Department, UIPSM.

Señale la opción más indicada para usted, de acuerdo a la siguiente escala:

0: Nada. **1: Algo.** **2: Moderadamente.** **3: Mucho.** **4: Muchísimo.**

1. Fumo para mantenerme despierto	0	1	2	3	4
2. Cuando fumo, me encuentro alerta, receptivo, atento	0	1	2	3	4
3. Me gusta fumar, cuando trabajo	0	1	2	3	4
4. Cuanto más cosas hago, más fumo	0	1	2	3	4
5. Si estoy preocupado por algo, fumo mucho...........................	0	1	2	3	4
6. Enciendo un cigarrillo, cuando estoy enfadado	0	1	2	3	4
7. Fumo, sin recordar haberlo encendido	0	1	2	3	4
8. Me encuentro fumando varios cigarrillos al mismo tiempo	0	1	2	3	4
9. Me encuentro a menudo encendiendo un cigarrillo por rutina, sin desearlo realmente	0	1	2	3	4
10. A menudo me pongo cigarrillos sin encender u otros objetos en la boca (bolígrafos, palillos, chicles, etc.) y los chupo para relajarme del estrés, tensión, frustración................................	0	1	2	3	4
11. Cuando fumo, me relaciono mejor con la gente	0	1	2	3	4
12. Fumo mucho más cuando estoy con otra gente	0	1	2	3	4
13. Cuando fumo, hablo más con la gente....................................	0	1	2	3	4
14. Algunos lugares o circunstancias me incitan a fumar [mi sillón favorito, sofá, habitación, coche o la bebida (alcohol, café, etc.)]	0	1	2	3	4
15. Cuando estoy sólo en un restaurante, parada de autobús, fiesta, etc., me siento más seguro, a salvo o más confiado con un cigarrillo en las manos..	0	1	2	3	4
16. Mi hábito de fumar es una parte importante de mi vida	0	1	2	3	4
17. Me recompenso a mí mismo con un cigarrillo tras cumplir una tarea...	0	1	2	3	4
18. Cuando no tengo tabaco, me resulta difícil concentrarme y realizar cualquier tarea	0	1	2	3	4
19. Juego y manipulo el cigarrillo como parte del ritual del hábito de fumar...	0	1	2	3	4
20. Me pongo algo en la boca para evitar fumar..........................	0	1	2	3	4
21. Cuando me hallo en un lugar en el que está prohibido fumar, juego con un cigarrillo o paquete de tabaco	0	1	2	3	4
22. Parte de mi placer de fumar procede del ritual que supone encender un cigarrillo	0	1	2	3	4
23. No sé mover las manos sin un cigarrillo.................................	0	1	2	3	4

STROBE Statement—checklist of items that should be included in reports of observational studies

	Item No.	Recommendation	Page No.	Relevant text from manuscript
**Title and abstract**	1	(*a*) Indicate the study's design with a commonly used term in the title or the abstract	1	
		(*b*) Provide in the abstract an informative and balanced summary of what was done and what was found	1	
**Introduction**				
Background/rationale	2	Explain the scientific background and rationale for the investigation being reported	2-3	
Objectives	3	State specific objectives, including any prespecified hypotheses	2-3	
**Methods**				
Study design	4	Present key elements of study design early in the paper	3-7	
Setting	5	Describe the setting, locations, and relevant dates, including periods of recruitment, exposure, follow-up, and data collection	3-7	
Participants	6	(*a*) *Cohort study*—Give the eligibility criteria, and the sources and methods of selection of participants. Describe methods of follow-up<br>*Case-control study*—Give the eligibility criteria, and the sources and methods of case ascertainment and control selection. Give the rationale for the choice of cases and controls<br>*Cross-sectional study*—Give the eligibility criteria, and the sources and methods of selection of participants	3-7	
		(*b*) *Cohort study*—For matched studies, give matching criteria and number of exposed and unexposed<br>*Case-control study*—For matched studies, give matching criteria and the number of controls per case		

Variables	7	Clearly define all outcomes, exposures, predictors, potential confounders, and effect modifiers. Give diagnostic criteria, if applicable	3-7
Data sources/ measurement	8*	For each variable of interest, give sources of data and details of methods of assessment (measurement). Describe comparability of assessment methods if there is more than one group	3-7
Bias	9	Describe any efforts to address potential sources of bias	3-7
Study size	10	Explain how the study size was arrived at	3-7

Quantitative variables	11	Explain how quantitative variables were handled in the analyses. If applicable, describe which groupings were chosen and why	3-7
Statistical methods	12	(*a*) Describe all statistical methods, including those used to control for confounding	3-7
		(*b*) Describe any methods used to examine subgroups and interactions	3-7
		(*c*) Explain how missing data were addressed	16
		(*d*) *Cohort study*—If applicable, explain how loss to follow-up was addressed<br>*Case-control study*—If applicable, explain how matching of cases and controls was addressed<br>*Cross-sectional study*—If applicable, describe analytical methods taking account of sampling strategy	
		(*e*) Describe any sensitivity analyses	
**Results**			
Participants	13*	(a) Report numbers of individuals at each stage of study—eg numbers potentially eligible, examined for eligibility, confirmed eligible, included in the study, completing follow-up, and analysed	7-8
		(b) Give reasons for non-participation at each stage	7-8

		(c) Consider use of a flow diagram	Figure 1
Descriptive data	14*	(a) Give characteristics of study participants (eg demographic, clinical, social) and information on exposures and potential confounders	7-8
		(b) Indicate number of participants with missing data for each variable of interest	7-8
		(c) *Cohort study*—Summarise follow-up time (eg, average and total amount)	
Outcome data	15*	*Cohort study*—Report numbers of outcome events or summary measures over time	
		Case-control study—Report numbers in each exposure category, or summary measures of exposure	
		Cross-sectional study—Report numbers of outcome events or summary measures	
Main results	16	(*a*) Give unadjusted estimates and, if applicable, confounder-adjusted estimates and their precision (eg, 95% confidence interval). Make clear which confounders were adjusted for and why they were included	7-8
		(*b*) Report category boundaries when continuous variables were categorized	7-8
		(*c*) If relevant, consider translating estimates of relative risk into absolute risk for a meaningful time period	

Other analyses	17	Report other analyses done—eg analyses of subgroups and interactions, and sensitivity analyses	
**Discussion**			
Key results	18	Summarise key results with reference to study objectives	8-12
Limitations	19	Discuss limitations of the study, taking into account sources of potential bias or imprecision. Discuss both direction and magnitude of any potential bias	11
Interpretation	20	Give a cautious overall interpretation of results considering objectives, limitations, multiplicity of analyses, results from similar studies, and other relevant evidence	8-12

Generalisability	21	Discuss the generalisability (external validity) of the study results	8-12
**Other information**			
Funding	22	Give the source of funding and the role of the funders for the present study and, if applicable, for the original study on which the present article is based	First page

*Give information separately for cases and controls in case-control studies and, if applicable, for exposed and unexposed groups in cohort and cross-sectional studies.

Note: An Explanation and Elaboration article discusses each checklist item and gives methodological background and published examples of transparent reporting. The STROBE checklist is best used in conjunction with this article (freely available on the Web sites of PLoS Medicine at http://www.plosmedicine.org/, Annals of Internal Medicine at http://www.annals.org/, and Epidemiology at http://www.epidem.com/). Information on the STROBE Initiative is available at www.strobe-statement.org.

© 2025 de Granda-Orive J.I. et al.
